# Opening Schools and Trends in SARS-CoV-2 Transmission in European Countries

**DOI:** 10.3389/ijph.2021.1604076

**Published:** 2021-08-18

**Authors:** Alessandra Buja, Federico Zabeo, Vittorio Cristofori, Matteo Paganini, Tatjana Baldovin, Riccardo Fusinato, Giovanna Boccuzzo, Silvia Cocchio, Silvia Coretti, Vincenzo Rebba, Maria Parpinel

**Affiliations:** ^1^ Department of Cardiologic, Vascular and Thoracic Sciences, and Public Health, University of Padova, Padova, Italy; ^2^ Department of Biomedical Sciences, University of Padova, Padova, Italy; ^3^ Department of Statistical Sciences, University of Padova, Padova, Italy; ^4^ Department of Economics and Management “Marco Fanno”, University of Padova, Padova, Italy; ^5^ Department of Medicine, DAME, University of Udine, Udine, Italy

**Keywords:** public health, COVID-19, pandemic, education, schools

## Abstract

**Objectives:** Benefits of school attendance have been debated against SARS-CoV-2 contagion risks. This study examined the trends of contagion before and after schools reopened across 26 countries in the European Union.

**Methods:** We compared the average values of estimated *R*_*t*_ before and after school reopening, identifying any significant increase with a one-sample *t*-test. A meta-analysis and meta-regression analysis were performed to calculate the overall increase in *R*_*t*_ for countries in the EU and to search for relationships between *R*_*t*_ before schools reopened and the average increase in *R*_*t*_ afterward.

**Results:** The mean reproduction number increased in 16 out of 26 countries. The maximum increase in *R*_*t*_ was reached after a mean 28 days. We found a negative relationship between the *R*_*t*_ before school reopening and its increasing after that event. By 45 days after the first day of school reopening, the overall average increase in *R*_*t*_ for the European Union was 23%.

**Conclusion:** We observed a significant increase in the mean reproduction number in most European countries, a public health issue that needs strategies to contain the spread of COVID-19.

## Introduction

A large-scale epidemic of COVID-19 caused by the severe acute respiratory syndrome coronavirus 2 has been occurring in Europe since January 2020 [1]. To mitigate community transmission of COVID-19, European governments introduced sweeping non-pharmaceutical interventions (NPIs) during the first and second waves of the epidemic. These measures have varied from one country to another, but have generally involved isolating confirmed cases, quarantine for contacts, social distancing, international and national travel restrictions, and the closure of schools and non-essential (or less-essential) shops and services [2].

The specific burden of each measure is hard to estimate because they have often been imposed, more or less simultaneously, and also because testing policies have changed over time [3].

It is important to understand the role of schools in the community transmission of SARS-CoV-2, bearing in mind that children and adolescents can spread the infection within families, even when their symptoms are mild [4].

Many countries and media reported clusters in preschool, primary and secondary schools [5–8].

The prevalence of confirmed cases of SARS-CoV-2 infection in schools seems to be influenced mainly by the level of transmission in the local community [9]. Transmission between children in the same class seems to be rare [10] due to the use of face masks, frequent hand washing, social distancing, and aeration. The risk of infection differs for different age groups, however. There is evidence of contagion rates usually being low in early childhood [11, 12], probably due to children being less susceptible to SARS-CoV-2 [13], while markedly higher rates are seen among adolescents.

In Italy in particular, many of the cases in the school-age population (40%) involved adolescents 14–18 years old, followed by primary-school children aged 6–10 (27%), middle-school children aged 11–13 (23%) and 3- to 5-year-olds attending preschool (10%) [5]. In France and Germany too, teenagers attending junior high school were the most often affected by outbreaks of the virus after schools reopened, and in Israel cases were reportedly more frequent in students 13–14 years old [14, 15].

Attending school involves groups having lessons together in classrooms for several hours, and other issues unconnected to face-to-face teaching, such as the use of public transport and eating meals, which need to be taken into account. Another important factor concerns social contact, which is known to be greater among adolescents, whose school attendance is often combined with other types of behavior that could facilitate the spread of viral infections, such as the use of public transport, meeting in bars or similar venues after lessons, and studying together. The contribution of this age group to the transmission of COVID-19 remains unclear, however.

In this study, we exploit daily data concerning the number of people newly infected with SARS-CoV-2 in European countries to investigate the possible temporal relationship between the reopening of schools after lockdowns and increase in the rate of the virus’s transmission.

## Methods

Data on the daily numbers of people testing positive for COVID-19 for each European country were obtained from https://ourworldindata.org/coronavirus– a coronavirus pandemic website that is updated daily. All data were collected in 2020, as of the first confirmed case of COVID-19 in each European country and up until 45 days afterwards. Spain has not provided data on the number of new cases of infection identified between Saturday and Sunday of each week since January 2020.

The dates when schools restarted in each European country were obtained from documents published by the Education, Audiovisual and Culture Executive Agency (EACEA) as part of its Education and Youth Policy Analysis (16), based on national data.

As the starting dates varied between different European countries, and sometimes also between different levels of education, and different regions of the same country, we considered the date when most of the schools started in a given country (Table 1).

**TABLE 1:** Day when most schools reopened in the various European countries. Opening schools and trends in SARS-CoV-2 transmission in European countries, Europe, 2020.

**European country**	**Day when schools reopened**
Austria	07/09/2020
Belgium	01/09/2020
Bulgaria	15/09/2020
Cyprus	04/09/2020
Croatia	07/09/2020
Denmark	10/08/2020
Estonia	01/09/2020
Finland	11/08/2020
France	11/09/2020
Germany	10/08/2020
Greece	14/09/2020
Ireland	07/09/2020
Italy	14/09/2020
Latvia	01/09/2020
Lithuania	01/09/2020
Luxemburg	15/09/2020
Malta	30/09/2020
Netherlands	17/08/2020
Poland	01/09/2020
Portugal	14/09/2020
Czechia	01/09/2020
Romania	14/09/2020
Slovakia	02/09/2020
Slovenia	01/09/2020
Spain	07/09/2020
Sweden	31/08/2020
Hungary	01/09/2020

As an indicator of the rate of the virus’s spread we chose the reproduction number, *R*_*t*_, defined as the average number of secondary infections caused by an individual who became infected at time *t*. Using this indicator (instead of the incidence of new cases every day) avoids the need to normalize the data in order to compare the results of analyses between different countries. It also takes into account the particular trend of the epidemic curve. We estimated *R*_*t*_ from the new daily number of people testing positive for COVID-19, using the algorithm developed by K. Systrom. To obtain comparable data, we applied a Gaussian weighted moving average filter with a 7-days window. We were unable to calculate the *R*_*t*_ for Latvia due to an unexplained error in the calculation process. Then, we compared the *R*_*t*_ for each day after the dates when schools reopened ($R_{t}^{i}$), with the average value of *R*_*t*_ estimated for the 7 days prior to schools reopening ($R_{t}^{\text{in}}$), adopting the approach used by Li et al. [17]:

$$R_{t}^{\text{in}}=\frac{1}{7}\overset{7}{\underset{i=1}{\sum }}R_{t}^{-i}$$

We calculated the *R*_*t*_ ratio ($R_{t}^{i}R$) for each day after the day schools reopened as:

$$R_{t}^{i}R=\frac{R_{t}^{i}}{R_{t}^{\text{in}}}$$

The *R*_*t*_ ratio can be interpreted as follows: the more $R_{t}^{i}R$ exceeds 1, the higher the reproduction number on the *i*th day after schools reopened with respect to the reference *R*_*t*_ prior to reopening. An $R_{t}^{i}R$ near 1 indicates a substantial parity between the two measures. To identify any significant mean increase in *R*_*t*_ in the days after schools reopened, we therefore conducted - for each country-a one-sample *t*-test (significance level = 0.05) to see whether the set of values {$R_{t}^{i}R,i=7,8,\cdots 42\}$ had an average significantly higher than 1.

**FIGURE 1:**
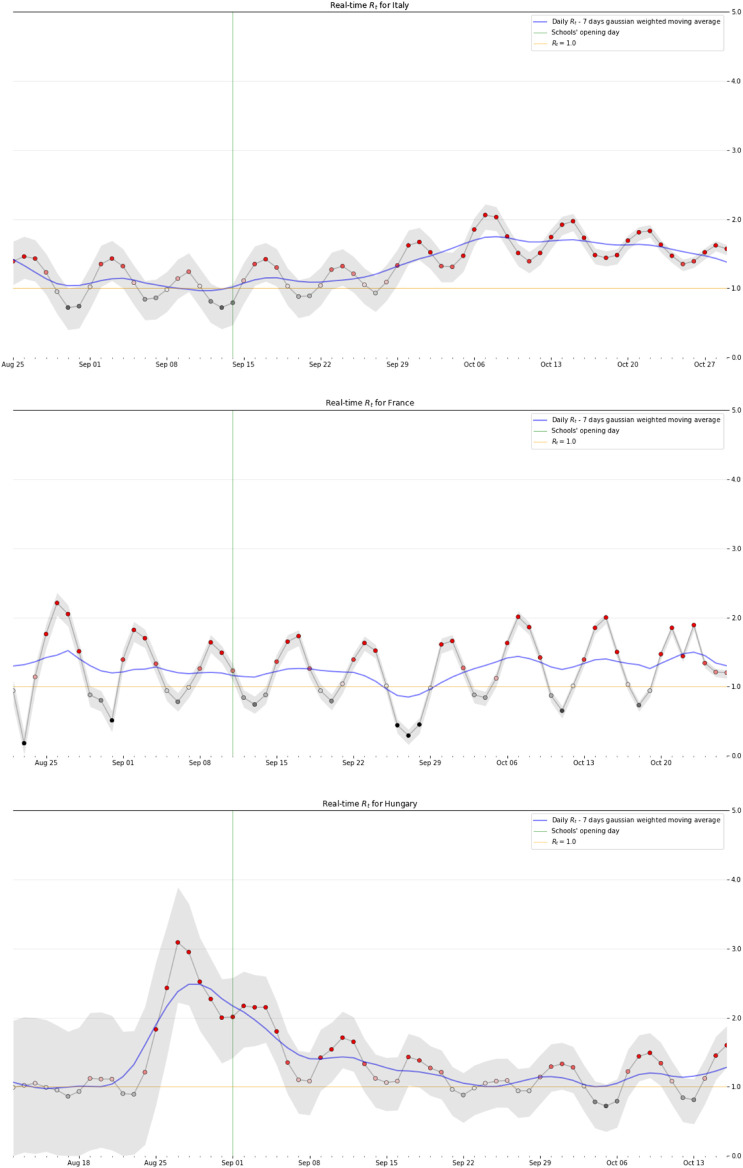
*R*_*t*_–90% Highest Density Interval (gray area)–of Italy, France and Hungary. Opening schools and trends in SARS-CoV-2 transmission in European countries, Europe, 2020.

In a second stage, we adopted a meta-analytic approach to pool the findings obtained for the 26 countries into a single result. We first weighted, then averaged the different results obtained. Since country-level behaviors were differed significantly (see Figure 1), we assumed that the average increase in *R*_*t*_ would differ from one country to another. We calculated the mean *R*_*t*_ ratio of each country, and weighted it with the variance for the country concerned (*V*_*i*_), and the variance among all European countries (*τ*). The weight of a country (*ω*_*i*_) was calculated as $\omega_{i}=\frac{1}{V_{i}+\tau^{2}}$.

Differences between the countries’ average rise in *R*_*t*_ can also be attributed to other hidden factors that were not considered in our analysis, such as the adoption of other preventive measures or the population’s adoption of a more or less virtuous behavior even after the easing of control measures. To partially address this issue, we investigated the association between the initial reference measure, and the average increase in *R*_*t*_ observed in the days after schools reopened. The relationship between the initial $R_{t}$ in each country and the average increase in *R*_*t*_ in the days after schools reopened was examined using a meta-regression analysis, which means a relationship between $R_{t}^{\text{in}}$ for a given country *j* and the weighted logarithm of the corresponding increase in $R_{t}$: ($ln\left(\bar{R_{t}R}\right)\times \omega_{j}$). $\bar{R_{t}R}$ was calculated as $\bar{R_{t}R}=\bar{R_{t}^{i}R\left(j\right)}=\frac{1}{35}\overset{42}{\underset{i=7}{\sum }}R_{t}^{i}R$ and $\omega_{j}$ was the weight obtained with the random effects meta-analysis.

## Results

We estimated the *R*_*t*_ (90% Highest Density Interval) during the first 6 weeks after the days on which schools reopened in the various European countries. Figure 1 shows examples of some types of slope.

**FIGURE 2:**
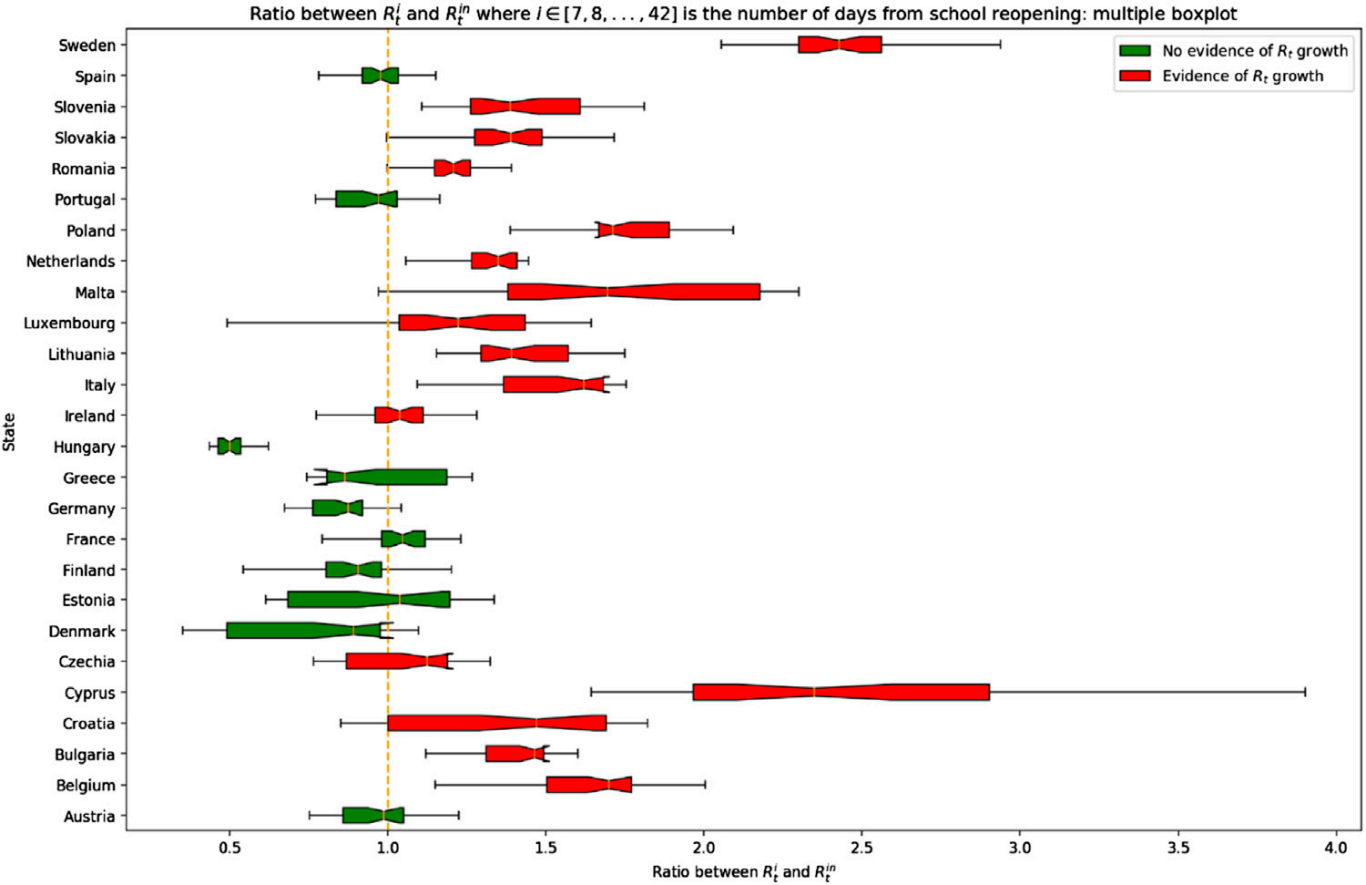
Average significant increase in reproduction number (*R*_*t*_ *)* compared to the days before schools reopened. Opening schools and trends in SARS-CoV-2 transmission in European countries, Europe, 2020.

Figure 2 shows the results of the $R_{t}^{i}R$ analysis.

An average $R_{t}^{i}R$ significantly higher than 1 was found in 16 out of 26 countries (61.5%). On average, the greatest increase in *R*_*t*_ in these countries was observed 28 days after schools reopened (95% CI: 22; 33).

**FIGURE 3:**
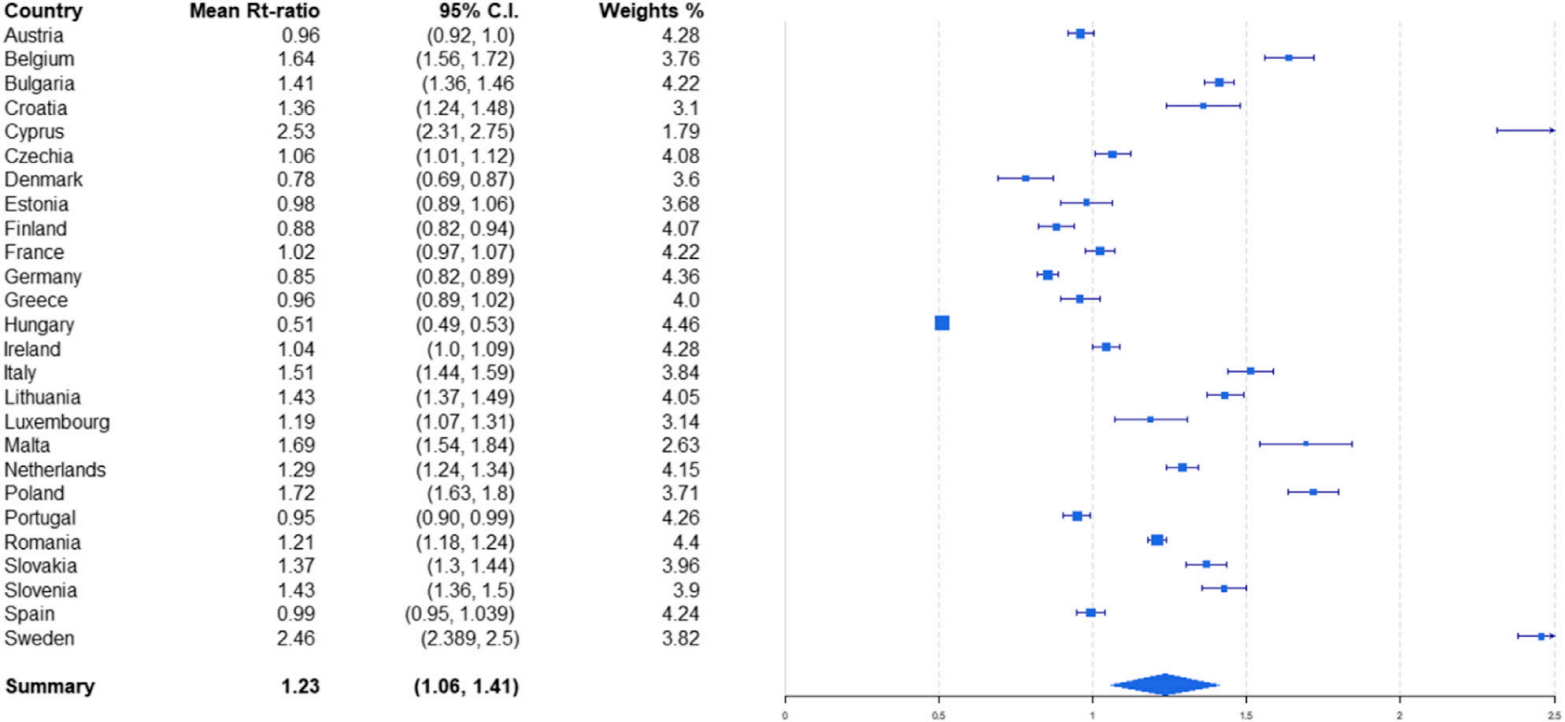
Mean reproduction number (R_t_)-ratio and weights for each country and overall. Opening schools and trends in SARS-CoV-2 transmission in European countries, Europe, 2020.

Figure 3 shows the weights and the results of the meta-analysis. We found the reopening of schools temporally correlated with a significant increase in the reproduction number, with an overall average increase of 23% (95% CI: 6%; 41%).

**FIGURE 4:**
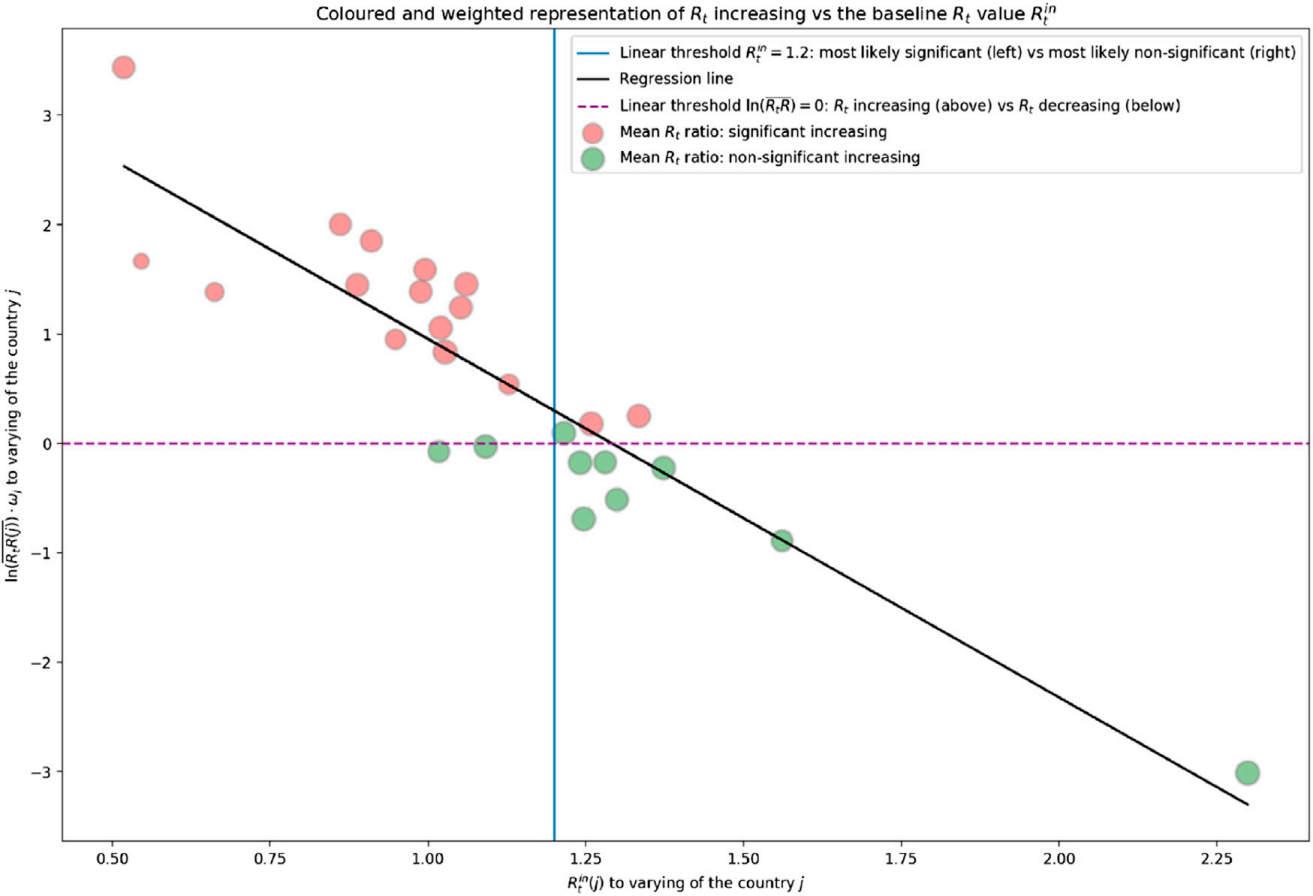
Regression line between the initial reproduction number ($R_{t}^{\text{in}}$) for a given country *j* and the weighted logarithm of its corresponding mean increasing ($ln\left(\bar{R_{t}R}\right)\times \omega_{j}$). Opening schools and trends in SARS-CoV-2 transmission in European countries, Europe, 2020.

The results of the meta-regression model are shown in Figure 4. The angular coefficient of the regression line was *m* = −3.278, and the intercept *q* = 4.233. The coefficient of determination *R* ^2^ was 0.83, and the MSE was 0.26.

These results suggest a significant negative correlation between the two variables: the higher the $R_{t}^{\text{in}}$ (the reproduction number before schools reopened, that was taken for reference), the lower the average increase in *R*_*t*_ after students returned to school. This might suggest that there was no significant increase prompted by schools reopening in those countries where the *R*_*t*_ was already very high. The graph in Figure 3 reveals a linear separator that accurately distinguishes between significant and non-significant increases, depending on the value of $R_{t}$. This is the line with the equation $R_{t}^{\text{in}}$ = 1.2. To the left of this line (i.e., for countries with $R_{t}^{\text{in}}<1.2$) we find 14 of the 16 countries where the *R*_*t*_ reportedly increased significantly, and only 2 of the 10 where this did not happen. To the right of the line, we find 8 of the latter 10 countries where *R*_*t*_ did not increase significantly after schools reopened and only 2 of the 16 countries where it did.

## Discussion

This study analyzed the trend of SARS-CoV-2 transmission before and after schools reopened in Europe.

We found a significant rise in the mean reproduction number from 7 to 42 days after schools reopened in 16 (Belgium, Bulgaria, Croatia, Cyprus, Czechia, Ireland, Italy, Lithuania, Luxemburg, Malta, Netherlands, Poland, Romania, Slovakia, Slovenia, Sweden) out of 26 European countries analyzed, with an overall 23% increase in the average *R*_*t*_ for the European Union compared with the days before students returned to school. This finding indicates that caution is needed in assessing the cost-benefit balance when designing lockdown loosening policies. National school closures to stem the COVID-19 pandemic had been implemented by 107 countries around the world by 18 March 2020, and in 192 countries by the end of April [2, 18]. The timing of their reopening has varied considerably across and within countries, and for different levels of education [19].

We found a significant negative correlation between the *R*_*t*_ prior to schools reopening and the mean increase in this value afterwards: the higher the *R*_*t*_ when schools reopened, the lower the increase in *R*_*t*_ over the 6 weeks thereafter. This might suggest that, in countries where there was no significant increase in the infection’s rate of spread after schools reopened, as in Hungary, this was because the *R*_*t*_ was already high and therefore unlikely to increase even further. Hungary also had the lowest ratio of swabs per 1,000 population of all European countries during the period of observation, so a number of new cases of infection may have gone undiagnosed [20].

A study analyzing the relative rates of infection by age group identified higher rates among adolescents and young adults than among children during the first wave of the epidemic in Germany [RR (age group): 0.78 [10–14]; 1.14 [15–19]; 1.40 [20–24]; 1.06 [25–27]] [21]. Another study conducted to identify sociodemographic factors behind the spread of COVID-19 found that provinces with lower aging indexes had higher rates of contagion, suggesting that younger people could be more responsible for spreading the virus at population level [22]. Our study identified no increase in $R_{t}^{i}R$ after schools reopened in France and Estonia, even though the numbers of new infections were still rising in these countries.

Our study has a major limitation that needs to be mentioned: we investigated changes in the $R_{t}^{i}R$ after schools reopened, but this does not imply a causal inference. “Before and after” analyses typically suffer from several internal validity issues, one of which may concern the effect of the passage of time: changes in an outcome measure might be due to some other influential event (s) occurring in the meantime. In our scenario, for instance, the reopening of sports facilities or seasonal temperature changes (which induce people to engage in different leisure activities) could have affected our outcome variable. In other words, the reopening of sports facilities, greater access to leisure activities, and a more frequent use of public transport may have contributed to the rise in disease transmission rates recorded after schools reopened [2]. We are confident nonetheless that our study contributes to the literature on the temporal dynamics of COVID-19, and can serve as a valid starting point for future research.

The management of school closures as a measure to contain the COVID-19 pandemic has represented a major challenge in European countries. Governments have faced a hard trade-off between safeguarding the population’s health and assuring young people’s education. In many cases, the reopening of schools during the pandemic was hotly criticized by the public. However, one-hundred-seven countries by March 18, 2020 and 192 countries by April 2020 implemented national school closures in response to the COVID-19 pandemic [2, 18]. The duration of school closures, and thus the timing of re-opening, has been very heterogeneous across countries and among school degrees within countries [19].

### Conclusion

Our study shows that reopening schools correlated with a faster diffusion of SARS-CoV-2 in most EU countries, and that countries with a low *R*_*t*_ at the time of schools reopening are more likely to see a marked rise of the incidence of infections afterwards. That said, school closures and, more broadly, the suspension of face-to-face education have negative consequences for students of various ages. As well as interfering with their academic progress, they can give rise to higher dropout rates, higher economic costs for families [23], and depression and anxiety in the young [24]. This means that various decision-making criteria and several different perspectives need to be taken into account when establishing policies relating to pandemic containment measures [25].

## Data Availability

The data underlying this article are derived from publicly available repositories. Please see methods for the details of each database used.
